# Differential expression of C5aR1 and C5aR2 in innate and adaptive immune cells located in early skin lesions of bullous pemphigoid patients

**DOI:** 10.3389/fimmu.2022.942493

**Published:** 2022-11-18

**Authors:** Shirin Emtenani, Maike M. Holtsche, Richard Stahlkopf, Daniel L. Seiler, Timothy Burn, Huiqing Liu, Melissa Parker, Kaan Yilmaz, Hasan O. Dikmen, Markus Huber Lang, Christian D. Sadik, Christian M. Karsten, Nina van Beek, Ralf J. Ludwig, Jörg Köhl, Enno Schmidt

**Affiliations:** ^1^ Lübeck Institute of Experimental Dermatology (LIED), University of Lübeck, Lübeck, Germany; ^2^ Department of Dermatology, Allergy, and Venereology, University of Lübeck, Lübeck, Germany; ^3^ Institute for Systemic Inflammation Research (ISEF), University of Lübeck, Lübeck, Germany; ^4^ Incyte Research Institute, Wilmington, DE, United States; ^5^ Institute of Experimental Trauma-Immunology, University Hospital of Ulm, Ulm, Germany; ^6^ Division of Immunobiology, Cincinnati Children’s Hospital Medical Centre, University of Cincinnati College of Medicine, Cincinnati, OH, United States

**Keywords:** autoimmune blistering disease, bullous pemphigoid, complement activation, complement component 5a receptor (C5aR) 1/2, neutrophils, C5a/C5aR axis

## Abstract

Bullous pemphigoid (BP), the by far most frequent autoimmune subepidermal blistering disorder (AIBD), is characterized by the deposition of autoantibodies against BP180 (type XVII collagen; Col17) and BP230 as well as complement components at the dermal-epidermal junction (DEJ). The mechanisms of complement activation in BP patients, including the generation of C5a and regulation of its two cognate C5aRs, i.e., C5aR1 and C5aR2, are incompletely understood. In this study, transcriptome analysis of perilesional and non-lesional skin biopsies of BP patients compared to site-, age-, and sex-matched controls showed an upregulated expression of *C5AR1*, *C5AR2*, *CR1*, and *C3AR1* and other complement-associated genes in perilesional BP skin. Of note, increased expressions of *C5AR2* and *C3AR1* were also observed in non-lesional BP skin. Subsequently, double immunofluorescence (IF) staining revealed T cells and macrophages as the dominant cellular sources of C5aR1 in early lesions of BP patients, while C5aR2 mainly expressed on mast cells and eosinophils. In addition, systemic levels of various complement factors and associated molecules were measured in BP patients and controls. Significantly higher plasma levels of C3a, CD55, and mannose-binding lectin-pathway activity were found in BP patients compared to controls. Finally, the functional relevance of C5aR1 and C5aR2 in BP was explored by two *in vitro* assays. Specific inhibition of C5aR1, resulted in significantly reduced migration of human neutrophils toward the chemoattractant C5a, whereas stimulation of C5aR2 showed no effect. In contrast, the selective targeting of C5aR1 and/or C5aR2 had no effect on the release of reactive oxygen species (ROS) from Col17-anti-Col17 IgG immune complex-stimulated human leukocytes. Collectively, this study delineates a complex landscape of activated complement receptors, complement factors, and related molecules in early BP skin lesions. Our results corroborate findings in mouse models of pemphigoid diseases that the C5a/C5aR1 axis is pivotal for attracting inflammatory cells to the skin and substantiate our understanding of the C5a/C5aR1 axis in human BP. The broad expression of C5aRs on multiple cell types critical for BP pathogenesis call for clinical studies targeting this axis in BP and other complement-mediated AIBDs.

## Introduction

Bullous pemphigoid (BP) is the most common subepidermal autoimmune blistering skin disease (AIBD) and primarily affects the elderly (1, 2). In central Europe and North America, the incidence is 13 to 42/million/year (3–7). In Northern Germany, the incidence of BP has recently been prospectively calculated to be 19.6 patients/million/year (8). BP is characterized and caused by autoantibodies against the hemidesmosomal BP180 (collagen type XVII, Col17) and BP230, which are expressed in basal keratinocytes abutting the dermal-epidermal/epithelial junction (DEJ) (1, 2). Clinically, BP typically presents with tense blisters, erosions, and urticarial plaques (9). Autoantibodies against Col17-NC16A and BP230 can be detected in the sera of approximately 70-90% and 50-60% of BP patients, respectively (10, 11) and deposit along the DEJ of skin and adjacent mucous membranes (12–15). A dense inflammatory infiltrate composed of mainly eosinophils and lymphocytes with accompanying macrophages and neutrophils is present in the upper dermis (16–18). The release of specific enzymes and reactive oxygen species (ROS) from granulocytes eventually leads to dermal-epidermal/epithelial separation (19–22).

Of note, the vast majority of BP patients exhibits C3c deposition along the DEJ (23), suggesting that complement-dependent pathway activation contributes to lesion formation. This view is supported by several studies in the neonatal mouse model of BP. In this model, complement activation, particular of the classical pathway, was shown to be essential for lesion formation (24–26). Accordingly, mutated non-C1q-binding anti-Col17 IgG1 was unable to induce skin lesions in neonatal COL17-humanized mice. In line, in an adult mouse model, C5-deficient mice developed only about half of skin lesions after injection of anti-Col17 IgG compared to wildtype animals (27, 28). In contrast to experimental models of BP, data about the relevance of complement activation in the human disease are rather scarce. In patients with BP, the intensity of C3 deposits in the skin and the capacity of sera to fix complement *in vitro* is well-established. In fact, the so-called complement fixation test correlated with disease activity (29, 30). In the same assay, the C3-fixing capacity of BP sera was abolished by addition of a C1s inhibitor (31). The same C1s inhibitor partially or completely abrogated C3c deposition at the DEJ in a phase I study in 4 of 5 BP patients (32).

Treatment of BP is still based on long-term use of systemic or superpotent topical corticosteroids that may be combined with potentially corticosteroid-sparing agents such as dapsone, doxycycline, methotrexate, azathioprine or mycophenoles (33–37). These regimens are associated with a high number of relapses and considerable adverse effects and are, in part, responsible for the increased mortality in BP (38–40). As such, there is a high medical need for safer and more effective treatment options for this fragile patient population (41, 42). Among the innovative treatment concepts, including inhibitors of IL-4R, IL-5R, IL-17, FcRn, and eotaxin, specifically targeting complement activation appears to be an attractive approach based on the data obtained in various mouse models of BP (16, 43–47).

To obtain insight into the complement system in human BP, we here comprehensively studied the complement activation in early skin lesions and in the blood of BP patients. We determined the expression pattern of C5aR1 and C5aR2 in early BP skin lesions and assessed systemic complement activation in plasma of BP patients. We also found strong upregulation of C5aR1 and C5aR2 in innate and adaptive immune cells as well as a functional role of autoantibody-mediated complement activation in this disease. Collectively, our data point toward an important role for C5aR1 activation in human BP which makes this receptor an attractive novel therapeutic target for this fragile patient population.

## Material and methods

### Human material

Sera, plasma, and skin samples from patients with BP, patients with non-inflammatory/non-infectious dermatoses dermatoses, and healthy individuals were collected at the Department of Dermatology at the University of Lübeck. The criteria for inclusion of BP patients were (i) compatible clinical picture without predominant mucosal involvement, (ii) linear deposits of IgG and/or C3c at the DEJ by direct IF microscopy of a perilesional biopsy, (iii) labelling of serum IgG at the epidermal side of 1 M-NaCl-split human skin by indirect IF microscopy, and (iv) circulating IgG against BP180-NC16A by ELISA (Euroimmun, Lübeck, Germany) or against LAD-1 by immunoblotting with conditioned concentrated medium of cultured HaCaT cells (48). Disease activity was measured by the bullous pemphigoid disease area index (BPDAI) (49). EDTA plasma, serum, and skin biopsies from BP patients were taken at the time of diagnosis before systemic therapy was initiated. All BP patients showed a classical BP phenotype with tense blisters and erosions. Since these samples are taken perilesionally and do not show fully developed lesions and as such, may reflect the early phase of the tissue destruction and inflammation, these samples were referred to as “early BP lesions” throughout the manuscript. This term does not mean to describe patients with non-bullous, premonitory, or urticarial BP. As control EDTA plasma, serum, and skin biopsies were taken from site-, age- ( ± 2 years), and sex-matched patients with non-inflammatory dermatoses dermatoses (mostly basal cell or squamous cell carcinoma). Polymorphonuclear leukocytes (PMNs) isolated from blood of healthy individuals was used for ROS release and chemotaxis assays. For the ROS release assay, immunoadsorption material of BP patients diagnosed as described above was employed. Sera and plasma were stored at -80°C until analyzed. For RNA sequencing skin samples were stored at -80°C. Paraffin embedded skin biopsies were utilized to perform immunohistochemistry analyses. Of note, two separate cohorts of BP patients were used: the RNA sequencing cohort (perilesional and non-lesional skin) and the immunohistochemistry cohort (perilesional skin). The studies were approved by the ethics committee of the University of Lübeck (18-046, 15-051, and 09-140) following the Declaration of Helsinki.

### RNA sequencing

To provide a detailed profile of complement activation in BP skin, mRNA expression of complement factors, complement receptors, and related molecules was analyzed by RNA sequencing. RNA of punch biopsies of perilesional BP skin (n=10), site-matched non-lesional BP skin (n=10) taken from the same patients at the same timepoint, and site-matched skin from age- and sex-matched patients with non-inflammatory dermatoses (n=10), subsequently referred to as control subjects, was isolated by InnuSPEED Tissue RNA kit (Analytik Jena, Upland, CA, USA) according to manufacturer’s instruction. The quality of total RNA was determined using Agilent 2100 Bioanalyzer system (Agilent Technologies, Santa Clara, CA, USA). Library preparation was performed by TruSeq^®^ stranded mRNA library preparation kit (Illumina, San Diego, CA, USA) using 1 µg of total RNA per sample. Samples were sequenced on an Illumina NextSeq500 by using 75-bp paired-end reads (Illumina). RNA sequencing data was analyzed using the OmicSoft Suite (Qiagen, Hilden, Germany) and aligned to the Human.B38 reference genome using the OmicsoftGenCode.V33 gene model. Principle component analysis was applied to assess data quality which was based on aligned reads with one healthy control sample being identified as an outlier and removed from the downstream analysis. Finally, differentially expressed genes were identified between the three samples groups using pairwise analysis with DESeq (OmicSoft) as described previously (50, 51).

### Immunohistochemistry

Expression of the highly differentially upregulated genes *C5AR1* and *C5AR2* was further studied on the protein level by immunohistochemistry. Punch biopsies of perilesional skin of BP patients (n=9) and controls (n=4) with non-inflammatory/non-infectious dermatoses matched for biopsy site, age, and sex were used. Here, perilesional skin was defined as skin without subepidermal splitting as verified by H&E-stained sections. Briefly, formalin-fixed, paraffin-embedded, 6-µm-thick tissue sections on Superfrost Plus™ slides (ThermoFisher Scientific, Dreieich, Germany) were deparaffinized in xylene and then dehydrated with graded ethanol series. Antigenicity was restored using heat-induced or proteolytic-induced epitope retrieval. For heat-antigen retrieval, sections were incubated in citrate buffer solution (pH 6.0) for 10 min in a pressure cooker. For enzymatic antigen retrieval, sections were subjected to pepsin digest-ALL 3 solution or proteinase K (both ThermoFisher Scientific) for 10 min at 37°C. Afterwards, slides were washed with PBS/0.05% Tween20 and blocked with 5% (v/v) normal donkey serum (Jackson ImmunoResearch Laboratories, Suffolk, UK) for 1 h at room temperature (RT). To identify the cellular site(s) of C5aR expression, we performed co-staining of rabbit anti-human C5aR1 (#PA5-32683, ThermoFisher Scientific) or C5aR2 (#PA5-33374, ThermoFisher Scientific) antibody with mouse anti-human myeloperoxidase (MPO; clone 392105, R&D Systems, Minneapolis, MN, USA) for neutrophils, mast cell tryptase (MCT; clone AA1, DAKO, Glostrup, Denmark) for mast cells, CD3 (clone F7.2.38, DAKO) for T cells, eosinophil peroxidase (EPX; clone MM25-82.2, Mayo Clinic, Scottsdale, AZ, USA) for eosinophils, and CD68 (clone PG-M1, DAKO) for macrophages. Following overnight incubation at 4°C, slides were washed and incubated with Alexa Fluor 594-AffiniPure donkey anti-rabbit IgG (Jackson ImmunoResearch Laboratories) and Alexa Fluor 488 goat anti-mouse IgG (ThermoFisher Scientific) for 1 h at RT. Slides were then washed and mounted with DAPI Fluoromount G (Southern Biotech, Birmingham, AL, USA). Normal rabbit IgG (Bio X Cell, Lebanon, NH, USA) and mouse IgG1, IgG2a, IgG2b, and IgG3 (all Biolegend, San Diego, CA, USA) served as controls. Images were acquired on a Keyence BZ-9000E series microscope (Keyence GmbH, Neu-Isenburg, Germany) and analyzed using a BZII analyzer (Keyence GmbH). Cell numbers were determined by counting fluorescent cells in relation to DAPI positive cells in 5 visual fields of 2 sections at 40-fold magnification.

The specificity of the C5aR2 antibody was evaluated using a synthetic C5aR2 peptide (peptides&elephants, Hennigsdorf, Germany). The synthetic peptide contains the amino acid sequence (RRLHQEHFPARLQCVVDYGGSSSTEN) of the immunogen used to generate the anti-C5aR2 antibody (#PA5-33374, ThermoFisher Scientific). Different amounts of the peptide (dose range, 0.1-50 µg) were first co-incubated with 10 ng of the anti-C5aR2 antibody for 3 h at RT. The antibody with and without the peptide was then used to stain randomly selected perilesional BP skin sections following the standard protocol. Isotype control antibody as well as C5aR2-specific antibody co-incubated with a non-relevant peptide (50 µg) served as controls.

### ELISA for complement and complement-related factors

EDTA plasma and serum from BP patients (n=10 plus one 6-month follow-up of 4 patients) and age- and sex-matched controls (n=10) was used to determine levels of complement and complement-related factors as well as the different complement pathways. Of note, plasma samples were stored at -80°C within 30 min after venipuncture. 9 of 10 BP patients, whose serum samples were used for ELISA in our study, showed C3c deposition along the dermal-epidermal junction. EDTA plasma samples were subjected to CD55 ELISA (Abcam, Milton, UK), C5b-9 ELISA (BD Biosciences, Franklin Lakes, CA, USA), C3a ELISA (Quidel, San Diego, CA, USA), C5a ELISA (DRG International, Springfield, NJ, USA), Factor H ELISA (R&D Systems Europe, Abingdon, UK), and Factor B ELISA (Abcam) according to the manufacturers’ instructions.

Activities of the classical, alternative, and mannose-binding lectin pathways were determined in serum by the corresponding Wieslab^®^ immunoassay following the manufacturer’s instructions (SVAR, Malmö, Sweden). In detail, the wells of the microtiter strips were coated with specific activators of the respective pathway. This in combination with the composition of sample dilution buffer and the level of patient serum dilution ensured that only the respective pathway was activated. During the incubation of the diluted patient serum, complement was activated by the specific coating. Wells were then washed and the amount of C5b-9 complex formed on the plate surface was detected with a specific alkaline phosphatase labelled antibody to the C5b-9 neoantigen formed during formation of the membrane attack complex. Absorbance was read at 450 nm (CD55, C5b-9, C3a, C5a, factor H, and factor B) and 405 nm (pathway assays) using a GloMax plate reader (Promega, Mannheim, Germany). In addition, the ELISA results were correlated with the patients’ BPDAI.

### Chemotaxis assay

The migration of human PMNs towards C5a was tested using 6.5 mm transwell plates with 3-µm pore inserts (Corning Inc., Kennebunk, ME, USA) as described previously (52) with the following modifications. Isolated PMNs from healthy volunteers were resuspended to a density of 6×10^6^ cells/ml in complete RPMI-1640 medium (RPMI-1640 containing 1% fetal calf serum, 2 mM L-glutamine, 100 U/ml penicillin, and 100 µg/ml streptomycin). The bottom wells were filled with 800 µl of complete RPMI-1640 medium containing recombinant C5a (Hycult Biotech, Uden, The Netherlands) at a final concentration of 12.5 nM. Thereafter, 200 µl of cell suspension were pre-incubated without or with C5aR (ant)agonists, including PMX53 (a C5aR1 antagonist, 10 µM) (53), P32 (a C5aR2 (ant)agonist, 100 µM) (54) or A8D^71-73^ (a C5aR1/C5aR2 double antagonist, 12.5 µM) (55) at 37°C for 5 min. Subsequently, cells were seeded on a transwell insert and incubated for 1 h at 37°C and 5% CO_2_. Afterward, non-migrated cells from the transwell insert and migrated cells from the bottom well were recovered separately. The number of migrated cells was determined by Cytek Aurora flow cytometer (Cytek Biosciences, Fremont, CA, USA). Finally, the percentage of chemotactic PMNs was calculated by dividing the number of migrated cells by the total number of recovered cells from the transwell insert and the respective bottom well. As negative control, isolated PMNs were seeded on a transwell insert without addition of C5a to the bottom well to correct for cells that passed the pores due to chemokinesis.

### Immune complex-induced reactive oxygen species release assay

A LumiTrackTM high binding 96-well-plate (ThermoFisher Scientific) was coated with immune complexes consisting of recombinant tetrameric form of BP180 NC16A (Euroimmun) at a final concentration of 5 µg/ml and 1:10 diluted BP immunoadsorption material. Human PMNs were purified from healthy individuals following the Polymorphoprep protocol (PROGEN Biotechnik, Heidelberg, Germany). After erythrocyte lysis and centrifugation, cells were resuspended in chemiluminescence medium (containing RPMI-1640 without phenol red, 1% fetal calf serum, 1 g/ml glucose, and 25 mM HEPES). Then, we seeded 200 µl of PMNs (with a density of 1×10^6^ cells/ml) in each well with or without C5aR (ant)agonists, including PMX53, P32 or A8D^71-73^ at final concentrations of 0.1-10 µM. PMX-53 and P32 were kindly provided by Dr. Trent Woodruff, University of Queensland, Australia. As negative controls, PMNs with or without antigen or antibody were used. After addition of luminol (Sigma-Aldrich, Hamburg, Germany) at a final concentration of 0.2 mM chemiluminescence was immediately measured by a luminescence reader (GloMax^®^ Discover System, Promega) for a period of ∼2 h at 37°C (56).

### Statistics

All data were analyzed and plotted using GraphPad Prism (Version 8, GraphPad Software, San Diego, CA, USA). All data are presented as mean ± standard error of the mean (SEM). For comparison of two groups, we used t-test. Unless indicated otherwise, a two-way ANOVA with Holm-Šídák’s multiple-comparisons test was performed to determine significance. Differences were considered as statistically significant at p-values of *, *p*≤ 0.05; **, *p* ≤ 0.01; and ***, *p* ≤ 0.001.

## Results

### Early skin lesions of BP patients comprise upregulated mRNA levels of complement factors and receptors including C5AR1 and C5AR2

**Figure 1:**
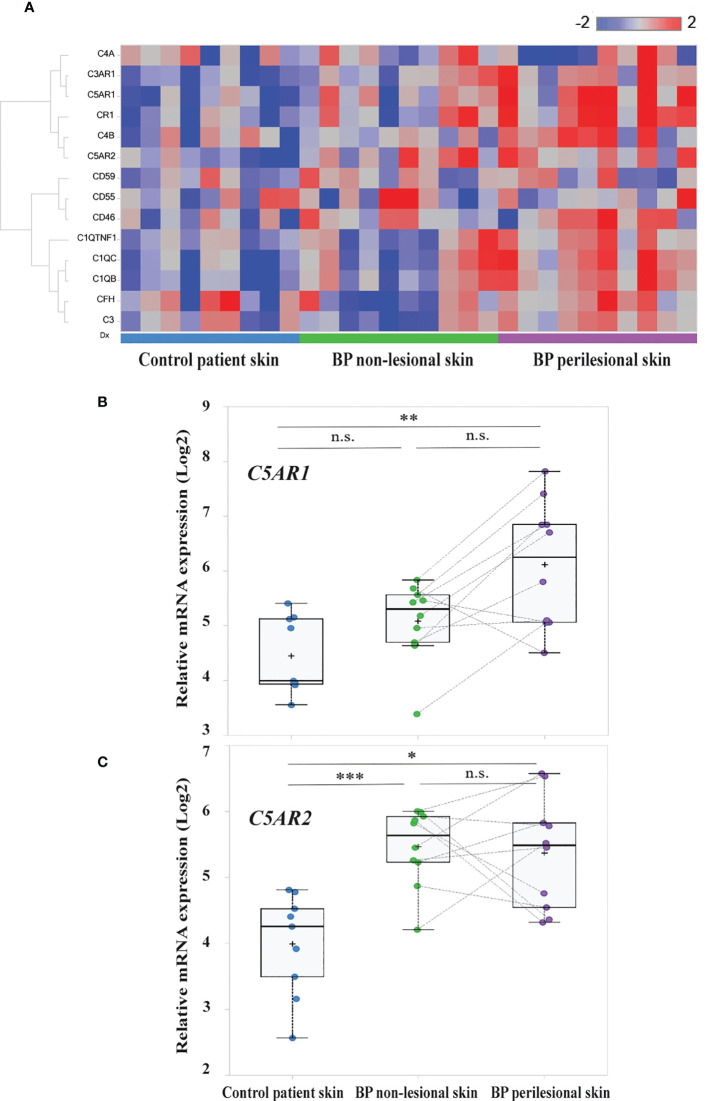
Transcriptome analysis identified *C5AR1* and *C5AR2* to be highly upregulated in early bullous pemphigoid (BP) skin lesions. **(A)** Heatmap of the complement and complement-related genes. RNA sequencing was performed on perilesional and site-matched non-lesional skin biopsies from BP patients (n=10) as well as site-matched biopsies from age- and sex-matched control subjects (n=9). Blue-red color bar: blue represents low gene expression and red high gene expression. **(B, C)** Box plots indicate the distribution of the relative mRNA expression levels of *C5AR1* **(B)** and *C5AR2* **(C)** in perilesional (purple) and site-matched non-lesional skin biopsies (green) from BP patients compared to the controls (blue). Plots were based on normalized and log2 transformed FPKM values and the identification of differentially expressed genes was conducted by DESeq2. FPKM, fragments per kilobase of transcript per million mapped reads; FDR, false discovery rate. *, FDR <0.05, **, FDR <0.01; **, FDR <0.001; n.s., not significant. , ***p ≤ 0.0001.

In order to unravel complement gene expression in BP patients’ skin RNA sequencing was performed in early BP skin samples, i.e., in biopsies from perilesional skin. Site-matched biopsies from non-lesional, i.e., clinically normally- appearing BP skin, and site-matched skin from age- and sex-matched controls with non-inflammatory/non-infectious dermatoses served as controls. Analysis of RNA sequencing data focused on complement and complement-related genes. Differentially expressed complement-related genes in (i) BP perilesional skin vs. control patient skin, (ii) BP non-lesional skin vs. control patient skin, and (iii) BP perilesional skin vs. BP non-lesional skin are shown in the heatmap of **Figure 1A**. Significantly elevated mRNA expression of *C5AR1* (false discovery rate (FDR), 0.0007) and *C5AR2* (FDR, 0.0035) were found between perilesional BP skin and control patient skin (**Figures 1B, C**). Significantly higher mRNA levels of *C5AR2* (FDR, 0.000093) were also seen in non-lesional BP skin samples compared to controls (**Figure 1C**).

We also detected significantly increased mRNA levels of other complement receptors as well as complement factors and associated proteins, including *CR1*, *C3AR1*, *C1QB*, *C1QC*, and *C1QTNF1* **(**FDR, 0.004; 0.0021; 0.0017; 0.0075, and 0.0078, respectively), in perilesional BP skin compared to site-matched skin of controls (**Figure S1**). No significantly elevated mRNA levels of *CD46* (FDR, 0.4412), *CD59* (FDR, 0.7226), and *CD55* (FDR, 0.9936) were found in early BP skin lesions compared to both non-lesional skin and skin of controls (**Figure S2**).

### In early skin lesions of BP patients, T cells and macrophages predominantly express C5aR1, whereas mast cells and eosinophils are the main sources of C5aR2 expression

**Figure 2:**
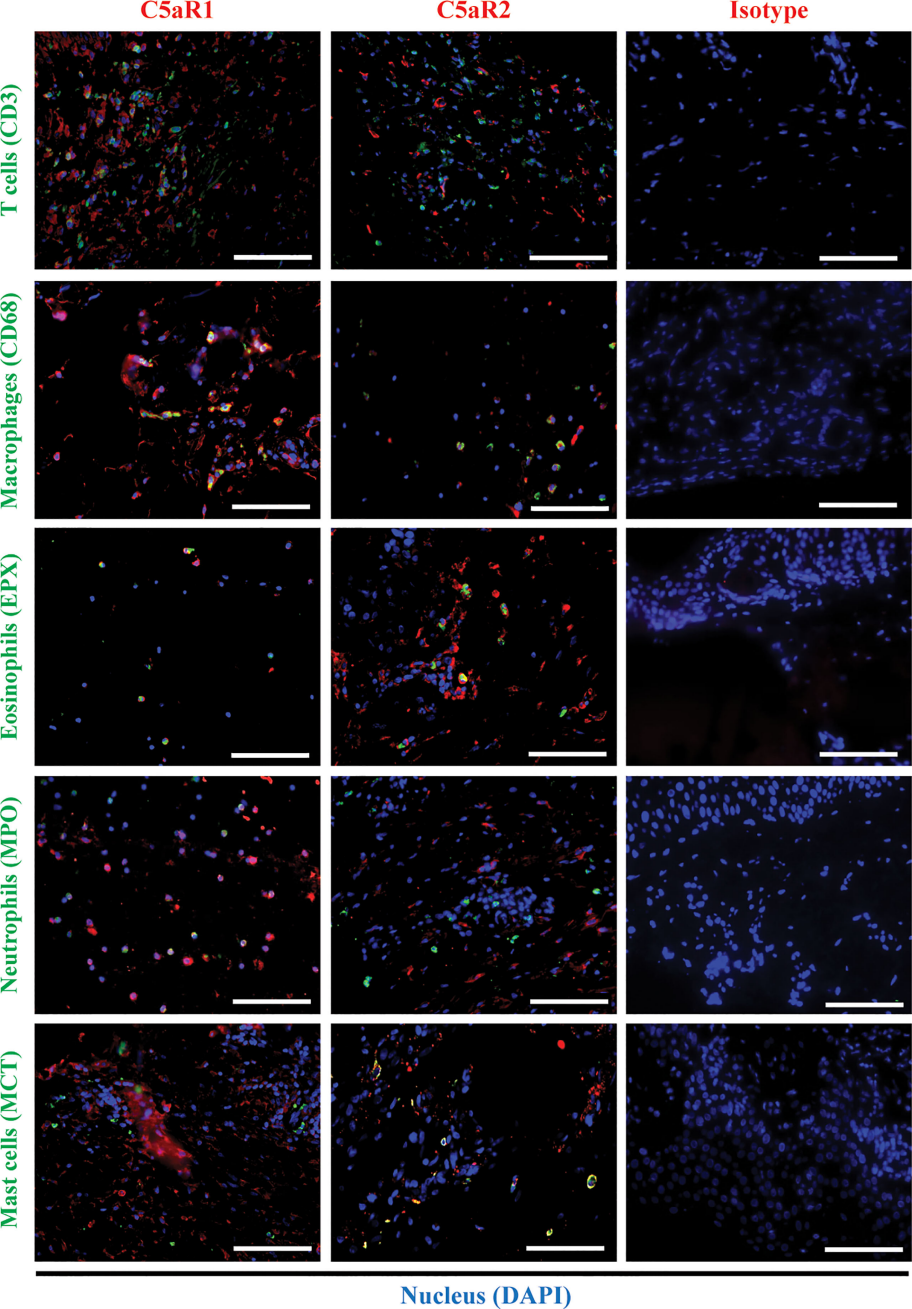
Double immunofluorescence (IF) staining revealed the cellular sources of C5aR1 and C5aR2 in early bullous pemphigoid (BP) skin lesions. IF staining on perilesional skin of BP patients (n=9) shows colocalization of C5aR1 (red) or C5aR2 (red) and cellular markers (green) on infiltrating T cells (CD3), macrophages (CD68), eosinophils (eosinophil peroxidase, EPX), neutrophils (myeloperoxidase, MPO), and mast cells (mast cell tryptase, MCT). Double positive cells appear in yellow. Stainings with isotype antibodies (Isotype) served as controls. DAPI staining of nuclei is shown in blue. Scale bars, 100 µm.

**Figure 3:**
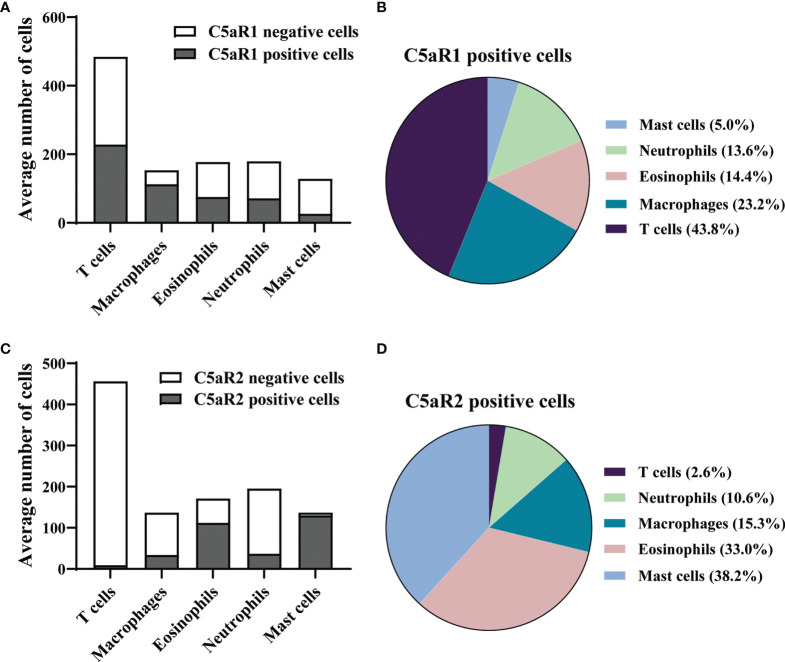
In early skin lesion of bullous pemphigoid (BP), T cells are the main source of C5aR1, while C5aR2 is predominantly expressed on mast cells and eosinophils. **(A, C)** Quantification of C5aR1- **(A)** and C5aR2-expressing cells **(C)** in perilesional BP skin samples (n=9) as determined by IF staining detailed in **Figure 2**. **(B, D)** Pie charts show the percentage of C5aR1- **(B)** and C5aR2-expressing cells **(D)** in relation to all inflammatory cell subsets in perilesional skin of BP patients.

To corroborate the RNA sequencing results at the protein level and identify the cellular sources of C5aR1 and C5aR2, immunohistochemical staining of C5aR1 and C5aR2 was performed in perilesional skin of BP patients and site-, age-, and sex-matched controls. In line with previous reports (16–18), the inflammatory infiltrate in BP skin lesions was dominated by T cells, eosinophils, neutrophils, and macrophages (17, 18). To map the expression sites of C5aRs, double-stainings of C5aR1 and C5aR2 on perilesional BP skin along with immune cell markers of these cells, i.e., CD3 (for T cells), eosinophil peroxidase (for eosinophils), myeloperoxidase (for neutrophils), CD68 (for macrophages) as well as mast cell tryptase (for mast cells) were performed (**Figure 2**). The highest frequency of C5aR1 expression was observed in macrophages (73%) and T cells (47%), respectively. The frequency of C5aR1 expression was lower in eosinophils (42%) and neutrophils (40%). Only 20% of mast cells stained positive for C5aR1 (**Figure 3A**). When C5aR1 expression was quantified in relation to the total C5aR1 expression of all inflammatory cells, T cells and macrophages appeared as the main cellular sources accounting for 43.8% and 23.2% of the total C5aR1 expression, respectively (**Figure 3B**). Besides, C5aR1 and C5aR2 expression was also detected on keratinocytes and endothelial cells (**Figure S3**).

Since only few studies have addressed the expression of C5aR2 in human tissues, we first set out to validate the specificity of the anti-C5aR2 antibody. When increasing amounts (0.1, 0.5, 1.0, 5, 25, and 50 µg) of the synthetic C5aR2 peptide, used to generate the anti-C5aR2 antibody, were added to the chosen dilutions of the anti-C5aR2 antibody, we found a dose-dependent reduction and a complete abolishment of C5aR2 staining at 25 µg and 50 µg C5aR2 peptide, confirming the C5aR2 specificity of the anti-C5aR2 antibody (**Figure S4**).

We found that 95% of mast cells and 65% of eosinophils stained positive for C5aR2, but only 25% of macrophages, 19% of neutrophils, and 2% of T cells (**Figure 3C**). Mast cells and eosinophils showed the highest contribution to the total C5aR2 expression with 38.2% and 33.0%, respectively (**Figure 3D**). As expected, skin samples of controls only contained few inflammatory cells and very lower numbers of C5aR1- or C5aR2-positive cells (**Figure S5**).

### BP patients exert elevated plasma levels of C3a, CD55, and components of the lectin pathway

After having addressed the local complement activation in the skin of BP patients, we subsequently studied the systemic complement activation by measuring classical-, alternative-, lectin- and terminal pathway activity as well as the anaphylatoxins C3a and C5a and some complement regulators in plasma of BP patients with active disease at the time of diagnosis. Plasma of age- and sex-matched patients with non-inflammatory skin diseases served as controls.

**Figure 4:**
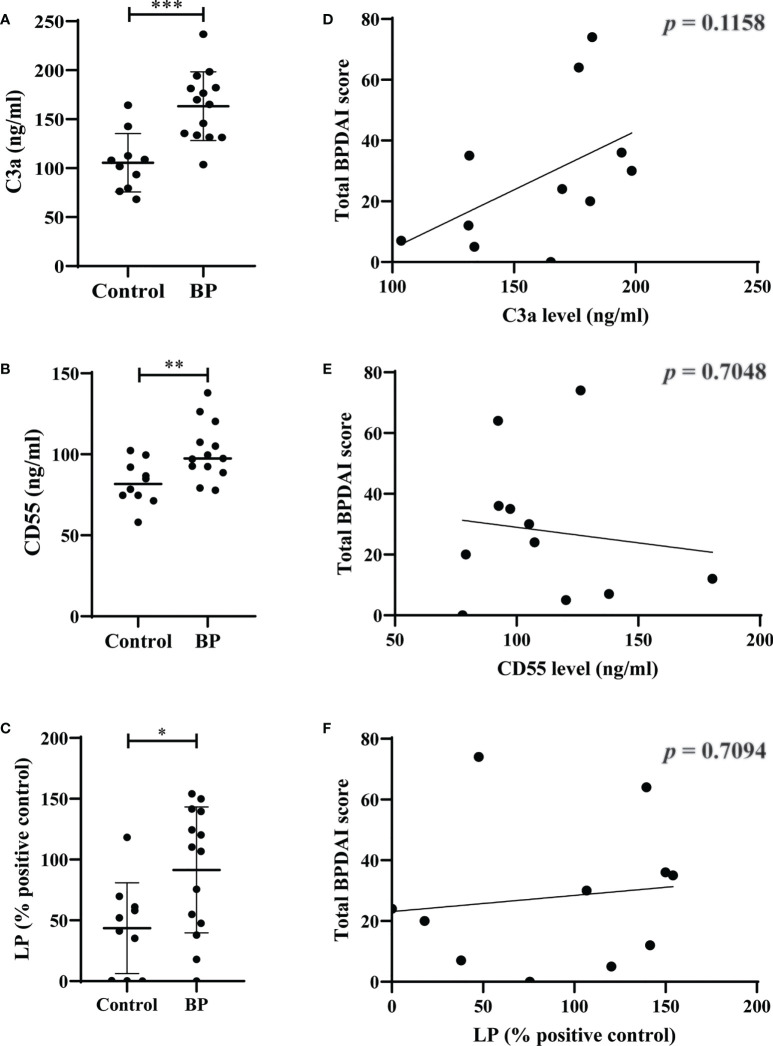
Elevated plasma levels of C3a and CD55 as well as elevated serum activity of mannose-binding lectin-pathway (LP) in patients with bullous pemphigoid (BP). **(A-C)** The plasma levels of C3a **(A)**, CD55 **(B)**, and serum activity of LP **(C)** in BP patients (n=10) were significantly increased compared to age- and sex-matched controls (n=10). **(D-F)** Plasma levels of C3a **(D)**, CD55 **(E)**, and serum activity of LP **(F)** in BP patients did not significantly correlate with disease activity as measured by the bullous pemphigoid disease area index (BPDAI). Differences between groups were analyzed by unpaired two-tailed t-test. *, *p* ≤ 0.05; **, *p* ≤ 0.01; ***, *p* ≤ 0.001.

We found significantly elevated plasma levels of C3a (*p=*0.0004) and CD55 (*p=*0.0091) as well as mannose-binding lectin-pathway activity (*p*=0.0208) in BP patients compared to controls (**Figures 4A-C**). In contrast, no significant differences were observed between plasma levels of C5a (*p*=0.3787), C5b-9 (*p*=0.1603), factor h (*p*=0.8148), factor b (*p*=0.2679), and the activity of the classical (*p*=0.1510) and alternative complement pathways (*p*=0.2526; **Figures S6A-F**). When plasma levels of the complement and complement-related factors as well as the pathway activities in BP patients were related with the BPDAI measured at the time when plasma was taken, no significant corrections were detected (**Figures 4D-F**, **S6G-L**).

### Pharmacological targeting of C5aR1 and/ or C5aR2 reduces chemotaxis of human neutrophils towards C5a

**Figure 5:**
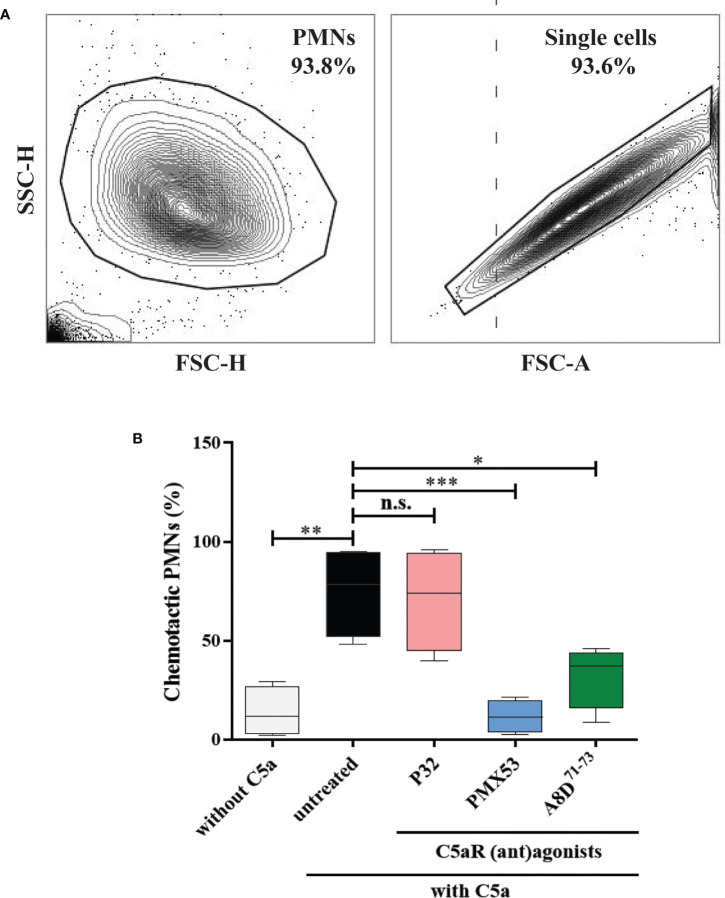
Pharmacological inhibition of C5aR1 significantly reduced the C5a-directed chemotaxis of normal human polymorphonuclear leukocytes (PMNs). **(A)** Flow cytometric gating strategy to identify human PMNs. Cells collected from the top insert and the bottom well of the transwells were pre-gated (area within the outline) using FSC-H vs. FSC-A to exclude cell debris, residual erythrocytes, and doublets. **(B)** Percentage of chemotactic PMNs in an *in vitro* migration assay towards C5a using transwell inserts. Chemotaxis of PMNs was induced by C5a in the presence of the C5aR2 agonist P32 (100 µM), the C5aR1 inhibitor PMX53 (10 µM), and the dual C5aR1/2 antagonist A8D^71-73^ (12.5 µM). Data were normalized to untreated cells. Cells not stimulated with C5a served as negative control. Results are compiled from four independent experiments with PMNs from different donors (n=4) and presented as mean ± SEM of migrated cells (percentage). Statistical analysis was performed using two-way ANOVA with Sidak’s multiple comparisons test. n.s., not significant; *, *p* ≤ 0.05; **, p ≤ 0.01, ***, p ≤ 0.001.

In mouse models of pemphigoid diseases, neutrophils critically contribute to tissue damage, and complement activation at the DEJ is a major driver for the infiltration of these cells into the skin (19, 20, 25, 57–59). While C5aR1 has been shown to exert a strong pro-inflammatory effect in these mouse models, both pro- and anti-inflammatory effects of C5aR2 have been reported in mouse models of BP and BP-like epidermolysis bullosa acquisita (28, 52, 59–61). Thus, we assessed the individual contribution of human C5aR1 and C5aR2 activation for C5a-dependent migration of polymorphonuclear granulocytes *in vitro* using cells from healthy donors (**Figure 5A**). Consistent with previous data obtained with mouse neutrophils (28, 52), the C5aR1 inhibitor PMX53 (53) markedly reduced the migration of the neutrophils towards C5a as compared with untreated cells (*p*=0.0008; **Figure 5B**), demonstrating a critical role for C5aR1 in C5a-mediated chemotaxis. Similarly, the C5aR1/C5aR2 dual antagonist A8D^71-73^ (55) significantly reduced C5a-mediated chemotaxis (*p*=0.0177; **Figure 5B**). To assess the individual contribution of C5aR2 to C5a-induced chemotaxis we next treated neutrophils with the C5aR2-specific agonist P32 (54). In contrast, the C5aR2 agonist did not impact on the C5a-driven chemotaxis (*p*=0.9935; **Figure 5B**), suggesting that the contribution of C5aR2 to C5a-mediated chemotaxis of human neutrophils is minor.

### Inhibition of C5aR1 or C5aR2 does not affect the Col17-anti-Col17 IgG immune complex-mediated ROS release from normal human leukocytes

**Figure 6:**
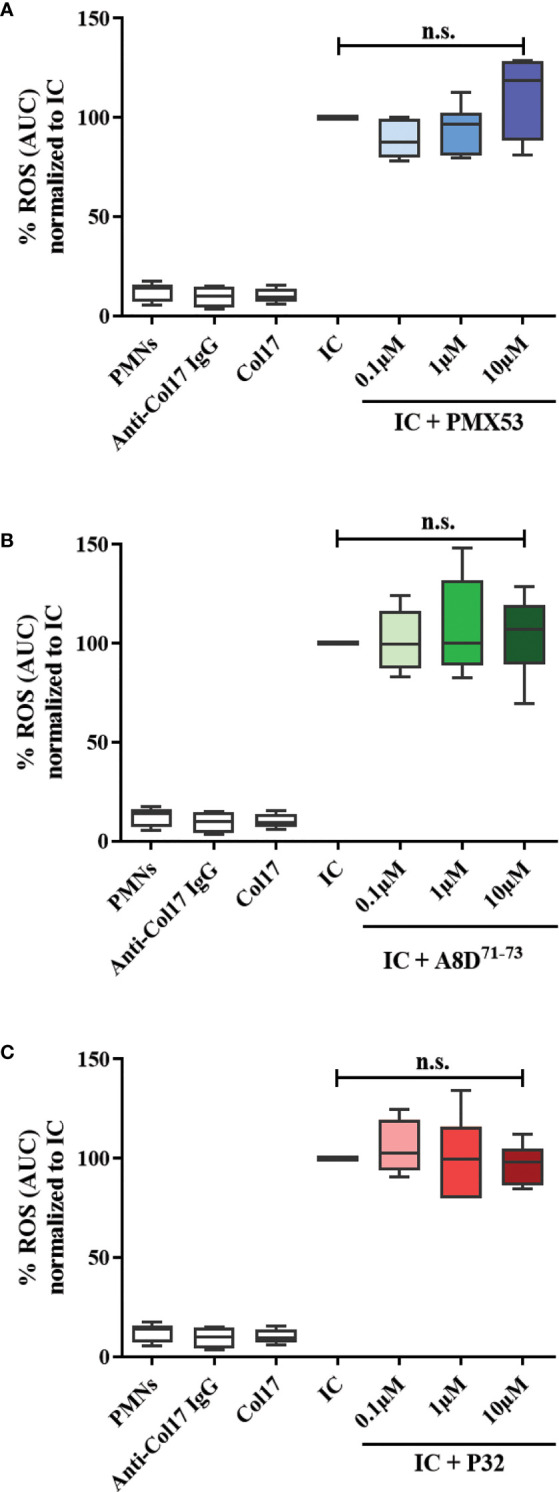
CaR1 and C5aR2 (ant)agonists have no effect on the reactive oxygen species (ROS) release from normal human polymorphonuclear leukocytes (PMNs) after stimulation with immune complexes (IC) of human Col17 and anti-Col17 IgG. PMNs of healthy volunteers (n=6) were activated with immobilized ICs of human Col17 and anti-Col17 IgG with or without the C5aR1 antagonist PMX53 **(A)**, the C5aR2 agonist P32 **(B)**, and the dual C5aR1/2 antagonist A8D^71-73^ **(C)** at three different concentrations (0.1, 1, and 10 µM). ROS release was tracked for 2 h and the AUC (cumulative values) of luminescence were calculated. Data were normalized to positive control (IC-stimulated PMNs). PMNs without or with antibody (anti-Col17 IgG) or antigen (Col17) served as negative controls. Results were pooled from six independent experiments with PMNs from different donors (n=6) and are presented as mean ± SEM. Data were analyzed using two-way ANOVA with Holm-Šídák’s multiple-comparisons test. n.s., not significant; AUC, area under curve.

Previous findings demonstrated bidirectional cross-talk between C5aR1 and FcγRs (62). To test a potential impact of C5aR1 on IgG immune complex-driven FcγR activation on human leukocytes, we determined the release of reactive oxygen species (ROS) from human leukocytes. This assay determines ROS release from human leukocytes in response to stimulation with immune complexes of recombinant human Col17 and human anti-Col17 IgG, mimicking leukocyte binding at the DEJ in BP patients. The C5aR1 inhibitor PMX53, the C5aR2 agonist P32, and the C5aR1/2 inhibitor A8D^71-73^ did not affect the IgG immune complex-driven ROS release of human leukocytes (**Figure 6**), suggesting that the ROS release from Col17-anti-Col17 IgG-stimulated human leukocytes occurs independently of the C5a/C5aR axis.

## Discussion

A convincing body of evidence for the pathogenic relevance of complement activation has previously been provided in various mouse models of pemphigoid diseases, including BP (24, 25, 27, 28, 60, 61, 63, 64). In particular, a central role of C5aR1 has been identified in these models (28, 60, 61, 65) supported by findings in other autoimmune disorders such as anti-myeloperoxidase glomerulonephritis, autoimmune uveitis, and psoriasis (66–68). The ample data about complement-mediated tissue destruction in mouse models of BP contrast with the scarcity of studies about the role of complement activation in patients suffering from BP. This is even more surprising since the labelling of C3c at the DEJ is a diagnostic hallmark of BP and found in 83-98% of patients (12, 23, 69, 70). The present study, therefore aimed at providing a detailed picture of local and systemic complement activation in BP patients and expression of complement receptors in skin lesions.

In an initial set of experiments, expression of complement factors in early BP skin lesions was studied by transcriptome analysis. Significantly higher mRNA levels of *C5AR1* and *C5AR2* were found in early BP skin lesions from perilesional skin biopsies compared to site-matched biopsies of age- and sex-matched controls. Furthermore, significantly higher mRNA levels of two other complement receptors, *CR1* and *C3AR1*, as well as the complement components *C1QB*, *C1QC*, and *C1QTNF1* were observed as compared with skin of control subjects. Of note, elevated expression of *C5AR2* (FDR, 0.000093, **Figure 1C**) and *C3AR1* (FDR, 0.0491, **Figure S1B**) was also observed in non-lesional BP skin compared to site-matched skin of controls. The latter results indicate that some components of the complement systems are activated even in macroscopically normal-appearing skin and may reflect an extremely early time point of skin inflammation shortly after IgG autoantibody binding to the DEJ. The relevance of upregulated expression of *C3AR1* in non-lesional BP skin is yet unclear. In light of recent findings which associated C3 upregulation in trigeminal ganglions with itch in a chemical-induced mouse model of allergic contact dermatitis, it is tempting to speculate that the early upregulation of *C3AR1* triggers itch sensation in BP (71), in particular as pruritus is present in nearly all BP patients and is not limited to areas with visible skin lesions (9). In line with this finding, elevated plasma levels of C3a were observed in BP patients in comparison to age- and sex-matched controls. Increased C3a levels have previously been observed in pruritic but not non-pruritic hemodialyzed patients (72). The previous finding that C3-deficient mice were susceptible to blister formation upon injection of anti-Col17 IgG argues against a direct contribution of this complement component in the development of visible skin inflammation and lesions of BP (73), but does not exclude its involvement in itch sensation. Interestingly, the expression level of *C5AR2* in non-lesional BP skin was similar to perilesional BP skin and significantly increased compared to site-matched skin of controls. Based on the anti-inflammatory effect of C5aR2 in the mouse model of BP (28) this may be interpreted as a counterregulatory mechanism to reduce C5aR1-mediated attraction of neutrophils. Indeed, anti-inflammatory mediators and cells including IL-10 and pro-resolving lipid mediators as well as regulatory T cells have already been described in BP (11, 74).

Furthermore, elevated mRNA levels of *C1QC*, *C1QB*, and *C1QTNF1* were observed in early BP skin lesions pointing towards a complex local network of activated complement factors in BP. This view is supported by data for complement regulatory proteins. These proteins regulate the enzymatic cascades, assembly of the membrane attack complex, and homeostasis of the complement system. Complement regulatory proteins include CD46 (membrane cofactor protein), CD59 (protectin), CD35 (CR1), and CD55 (decay accelerating factor) (75, 76), among others. Dysregulation of complement regulatory proteins directly affects the progression of several autoimmune diseases, such as systemic lupus erythematosus and rheumatoid arthritis (77). Here, we revealed increased mRNA levels of *CR1* but not of *CD46*, *CD55*, and *CD59* in perilesional BP skin. CR1 exerts a dual function as a phagocytic receptor for C3b-opsonized pathogens and a regulator of the C3/C5 convertases and co-factor for factor I to cleave C3b into iC3b, C3c, and C3dg. Its upregulation may point toward a counter-regulatory measure to control the amplification loop of the alternative pathway at the DEJ, where IgG immune complexes have bound and activated the complement cascade. Previous studies in BP reported downregulated *CD55* expression (75), whereas CD46 levels were significantly enhanced in sera and blister fluids of BP patients, but its mRNA level was downregulated in BP skin lesions (78).

The complement genes with the most striking difference in mRNA expression between early BP skin lesions and skin of control subjects were *C5AR1* and *C5AR2*. The anaphylatoxin C5a exerts its effector functions through binding to its two receptors, namely C5aR1 (CD88) and C5aR2 (GPR77, C5L2) (79). C5aR1 exerts a proinflammatory role in several autoimmune diseases, whereas the role of C5aR2 is still enigmatic, with both immune-activating and immunosuppressive functions in inflammatory disease models such as allergic contact dermatitis and allergic asthma (80–84). Therefore, we subsequently studied the expression of C5aR1 and C5aR2 in early BP skin lesions, i.e., skin biopsies taken directly adjacent to a blister or erosion but without microscopic split formation, by immunohistochemistry. Strong expression of both complement receptors were observed in early BP lesions compared to site-matched skin of age- and sex-matched controls. To address whether the increased expression of C5aR1 and C5aR2 is due to increased expression of the receptors on individual cells or rather increased cell numbers, the percentage of C5aR1 and C5aR2 expression cells was calculated. By double immunohistochemistry, we identified T cells and macrophages as the dominant cell types expressing C5aR1 and mast cells and eosinophils as the main cell types expressing C5aR2. Our findings align with the previous observation that C5aR1 and C5aR2 are expressed on human monocytes, but contrast with Arbore et al., who reported resting and activating T cells to preferentially express C5aR2 and only to a low extent C5aR1 *in vitro* (82, 85). In skin lesions of BP patients, T cells are the main producers of IL-17A (16–18, 86), a cytokine that has been shown to be essential for blister formation in the antibody transfer adult mouse model of BP (16). Macrophages, mast cells, and eosinophils are pivotal for lesion formation in the neonatal and local mouse models of BP, respectively (65, 87, 88). The importance of C5aR1 on mast cells for blister formation has been described in the neonatal mouse model of BP (65), however may be questioned for the human disease, since in the present study only 20% of mast cells expressed C5aR1 and mast cells only contributed to about 5% of C5aR1 expression in early BP lesions. In addition, expression of C5aRs was also detected on keratinocytes and endothelial cells. Induction of C5aR1 mRNA in keratinocytes under different inflammatory conditions has previously been described (89). A more recent study reported high expression of C5aR1 on keratinocytes in perilesional BP skin without further investigating the functional role of C5aR1 on these cells (45).

In addition to delineating the complex network of complement activation in early skin lesions of BP patients, we were interested in the systemic complement activation in BP patients. We found elevated plasma levels of C3a, CD55, and lectin pathway activity compared to age- and sex-matched controls. These data are in agreement with a previous report of the significant correlation of sCD46 and C3a in BP sera (78). In contrast, another study failed to show elevated plasma levels of C3a in BP patients (31). This discrepancy may be explained by our effort to freeze all BP plasma samples within 30 min after venepuncture. The lack of correlation between plasma levels of C3a, CD55, and lectin pathway activity with disease activity as measured by BPDAI leads us to conclude that local complement activation in the skin rather than in the circulation is of pathogenic relevance in patients with BP. The discrepant findings of the elevated levels of systemic C3a without a parallel increase of C5a and C5b-9 may be explained by the notion that C3a plasma levels are generally much higher than C5a levels. The rapid degradation process as well as the about 100-fold lower C5a levels compared to C3a levels render finding significantly different C5a levels in BP per se more difficult. Although BP is known to be mainly driven by the classical pathway (25, 27), we here observed an activation of the lectin pathway, whereas no significant activation of the classical pathway was found. Of note, the participants in the current study have already established BP. Hence, it is conceivable that, as shown in mice, the classical pathway of complement activation is the disease-initiating pathway, while the lectin pathway may serve as driver of continued complement activation during established disease. Clearly, further research will be necessary to clarify the role of lectin pathway activation in the development of BP.

It has been demonstrated that C5a initiates inflammation not only through its role as a cell activator and chemoattractant but also *via* its effects on FcγRs, suggesting an intriguing crosstalk between C5a and FcγR. Using an acute immune complex pulmonary hypersensitivity model, C5aR activation was found to be necessary to initiate neutrophil recruitment and a proinflammatory FcγR response (90, 91). Moreover, interaction between neutrophilic C5aR and FcγRIIa was shown to be essential for disease progression in a humanized mouse model of inflammatory arthritis (92). In the last two sets of experiments, we addressed the functional relevance of complement activation and its pharmacological targeting in two well-established *in vitro* assays (52, 56). It is known from mouse models of pemphigoid diseases that neutrophils play an important role in the pathogenesis of these diseases, particularly by releasing ROS (28). Hence, we here analyzed the effect of C5a-C5aR interaction on neutrophils as one of the main producers of ROS. A specific inhibitor of C5aR1, a dual inhibitor of C5aR1 and C5aR2, and a C5aR2 agonist did not alter the ROS release from normal human leukocytes after stimulation with human Col17-anti-Col17 IgG immune complexes. These results indicate that ROS release from immune complex-stimulated human leukocytes occurs independently of the C5a/C5aR axis. Besides, a previous study demonstrated that ROS production by eosinophils, detected here as the main cellular source of C5aR2, also requires FcγR activation (93). Therefore, it also possible that eosinophils may release ROS independently of the C5a/C5aR interaction, similar to neutrophils. Even though neutrophils did not appear as main source of C5aR expression, C5a generation from immune complex-stimulated neutrophils and C5a-mediated priming effects *via* C5aRs may allow neutrophils to generate ROS in response to immune complexes.

Subsequently, we demonstrated that the C5aR1 inhibitor and the dual C5aR1/C5aR2 inhibitor significantly reduced the chemotaxis of human neutrophils towards C5a, while no effect was seen with the C5aR2 agonist. These findings are in line with previous data obtained with cells from C5aR1- and C5aR2-deficient mice (28). Of note, neutrophils from C5aR2-deficient mice showed a decreased chemotaxis towards C5a, a finding that aligns with the reduced disease activity observed in C5aR2-deficient mice in the passive transfer mouse model of EBA, while in the passive transfer mouse model of BP, C5aR2-deficient mice developed significantly more skin lesions (28, 52). This discrepancy may be explained, at least in part, by different Fcγ receptors used in these models. In experimental BP, tissue destruction is mediated by FcγRIV and FcγRIII, whereas in the antibody transfer mouse model of EBA, it is restricted to FcγRIV (94, 95).

Collectively, our study highlights the complex network of complement activation in early BP skin lesions with upregulation of several complement factors, most strikingly of the two C5a receptors C5aR1 and C5aR2. Pathogenic relevant complement activation in BP primarily occurs in the skin and not in the circulation. Our neutrophil-based functional data suggest a minor contribution of C5aR2 to C5a-driven chemotaxis of cells; therefore, inhibition of C5aR1, in particular will be a promising therapeutic strategy for moderate and severe BP. Considering the complex network of complement activation in BP and the recent findings by Seiler *et al.,* in the antibody-transfer mouse model of epidermolysis bullosa acquisita (52), it is well possible that C5aR2 also has a relevant role in the initiation of tissue destruction in BP. As such, the successful phase III study and the recent FDA-approval of the C5aR1 inhibitor avacopan in ANCA-associated vasculitis (96) and a promising phase II study with the LTB4/C5a inhibitor nomacopan in BP (unpublished), that led to the initiation of a phase III trial, may pave the way for effective complement-related therapies for this disease.

## Data availability statement

The datasets presented in this study can be found in online repositories. The names of the repository/repositories and accession number(s) can be found below: BioProject, PRJNA900420. Requests to access the datasets may be addressed to the corresponding author(s).

## Ethics statement

This study was reviewed and approved by Ethikkommission der Universität zu Lübeck Ratzeburger Allee 160 Haus 2 23562 Lübeck. The patients/participants provided their written informed consent to participate in this study.

## Author contributions

SE, CK, JK and ES contributed to the study design. SE, MH, DS and RS performed the experiments. TB and HL analyzed the transcriptome data. ML carried out the ELISA assays. SE and ES wrote the manuscript. MH, KY, HOD and NB recruited patients and samples. All authors contributed to the article and approved the submitted version.

## Funding

This work was supported by grants from the Deutsche Forschungsgemeinschaft through CRU 303 *Pemphigoid Diseases*, CRC 1526 *Pathomechanisms of Antibody-mediated Autoimmunity*, and the Excellence Cluster 2167 *Precision Medicine in Chronic Inflammation* as well as an unrestricted research grant from Incyte (to RL and ES). The funder was not involved in the study design, collection, analysis, interpretation of data, the writing of this article or the decision to submit it for publication.

## Acknowledgment

We thank Vanessa Krull and Sylvana Schult for excellent technical assistance.

## Conflict of interest

Authors TB, HL and MP are employees and/or shareholders of Incyte Corporation.

The remaining authors declare that the research was conducted in the absence of any commercial or financial relationships that could be construed as a potential conflict of interest.

## Publisher’s note

All claims expressed in this article are solely those of the authors and do not necessarily represent those of their affiliated organizations, or those of the publisher, the editors and the reviewers. Any product that may be evaluated in this article, or claim that may be made by its manufacturer, is not guaranteed or endorsed by the publisher.

## Supplementary material

Supplementary Figure 1 mRNA levels of complement receptor and complement-associated genes are enhanced in early bullous pemphigoid (BP) skin lesions. (A-E) Dot-plot graphs depicting the relative RNA expression levels of differentially expressed genes, including *CR1* **(A)**, *C3AR* **(B)**, *C1QB* **(C)**, *C1QC* **(D)**, and *C1QTNF1* **(E)** between the three clinical groups: site-matched skin of age- and sex-matched controls (n=9), site-matched BP non-lesional skin (n=10), and BP perilesional skin (n=10). Plots were based on normalized and log2 transformed FPKM values and the identification of differentially expressed genes was conducted by DESeq2. FPKM, fragments per kilobase of transcript per million mapped reads; FDR, false discovery rate; *, FDR <0.05; n.s., not significant.

Supplementary Figure 2 mRNA levels of *CD46, CD59*, and *CD55* are not significantly altered in early bullous pemphigoid (BP) skin lesions compared to both non-lesional BP skin and skin of controls. **(A-C)** Dot-plot graphs depicting the relative mRNA levels of complement regulatory genes, including *CD46* (A), *CD59* **(B)**, and *CD55* **(C)** between the three clinical groups: site-matched skin of age- and sex-matched controls (n=9), site-matched BP non-lesional skin (n=10), and BP perilesional skin (n=10). Plots were based on normalized and log2 transformed FPKM values and the identification of differentially expressed genes was conducted by DESeq2. FPKM, fragments per kilobase of transcript per million mapped reads; FDR, false discovery rate; *, FDR <0.05; n.s., not significant.

Supplementary Figure 3 Immunofluorescence staining showed expression of C5aR1 **(A)** and C5aR2 **(B)** on epithelial cells and vascular endothelial cells. DAPI staining of nuclei is shown in blue. Scale bars, 100 µm.

Supplementary Figure 4 The specificity of the anti-C5aR2 antibody was validated by coincubation with increasing amounts of a C5aR2 peptide. Sections of randomly selected BP skin were stained with isotype control antibody **(A)**, anti-C5aR2 antibody alone **(B)**, and anti-C5aR2 antibody plus increasing amounts of the C5aR2 peptide (RRLHQEHFPARLQCVVDYGGSSSTEN), i.e. 0.1 **(C)**, 0.5 **(D)**, 1 **(E)**, 5 **(F)**, 25 **(G)**, and 50 µg **(H)**, respectively. A dose-dependent inhibition of the IF staining was seen with the increasing C5aR2 peptide amounts indicating a high specificity of the anti-C5aR2 antibody. Incubation of the anti-C5aR2 antibody with 50 µg of an irrelevant control peptide did not alter the IF staining (data not shown). Data are representative of results obtained from three experiments. Scale bars, 100 µm.

Supplementary Figure 5 In site-matched skin biopsies of age- and sex-matched control patients (n=4), only few inflammatory cells were detected with low expression of C5aR1 **(A)** and C5aR2 **(B)**.

Supplementary Figure 6 Plasma levels of C5b-9, C5a, factor h, and factor b as well as serum activity of the classical (CP) and alternative complement (AP) pathways in patients with bullous pemphigoid (BP) and controls. **(A-F)** Plasma levels of C5b-9 **(A)**, C5a **(B)**, factor h **(C)**, and factor b **(D)** as well as serum activity of the CP (E) and the AP **(F)** in BP patients (n=10) and age- and sex-matched controls (n=10) did not show significant differences. **(G-L)** Plasma levels of C5b-9 **(G)**, C5a **(H)**, factor h **(I)**, and factor b **(J)** as well as serum activity of the CP **(K)** and the AP **(L)** in BP patients did not significantly correlate with diseases activity as measured by the bullous pemphigoid disease area index (BPDAI). Differences between groups were analyzed by unpaired two-tailed t-test. n.s., not significant.
