# Supplementary figures and images for: Differential expression of C5aR1 and C5aR2 in innate and adaptive immune cells located in early skin lesions of bullous pemphigoid patients
Source: Front Immunol. 2022 Nov 18;13:942493. doi: 10.3389/fimmu.2022.942493 (PMC9716273; doi:10.3389/fimmu.2022.942493)

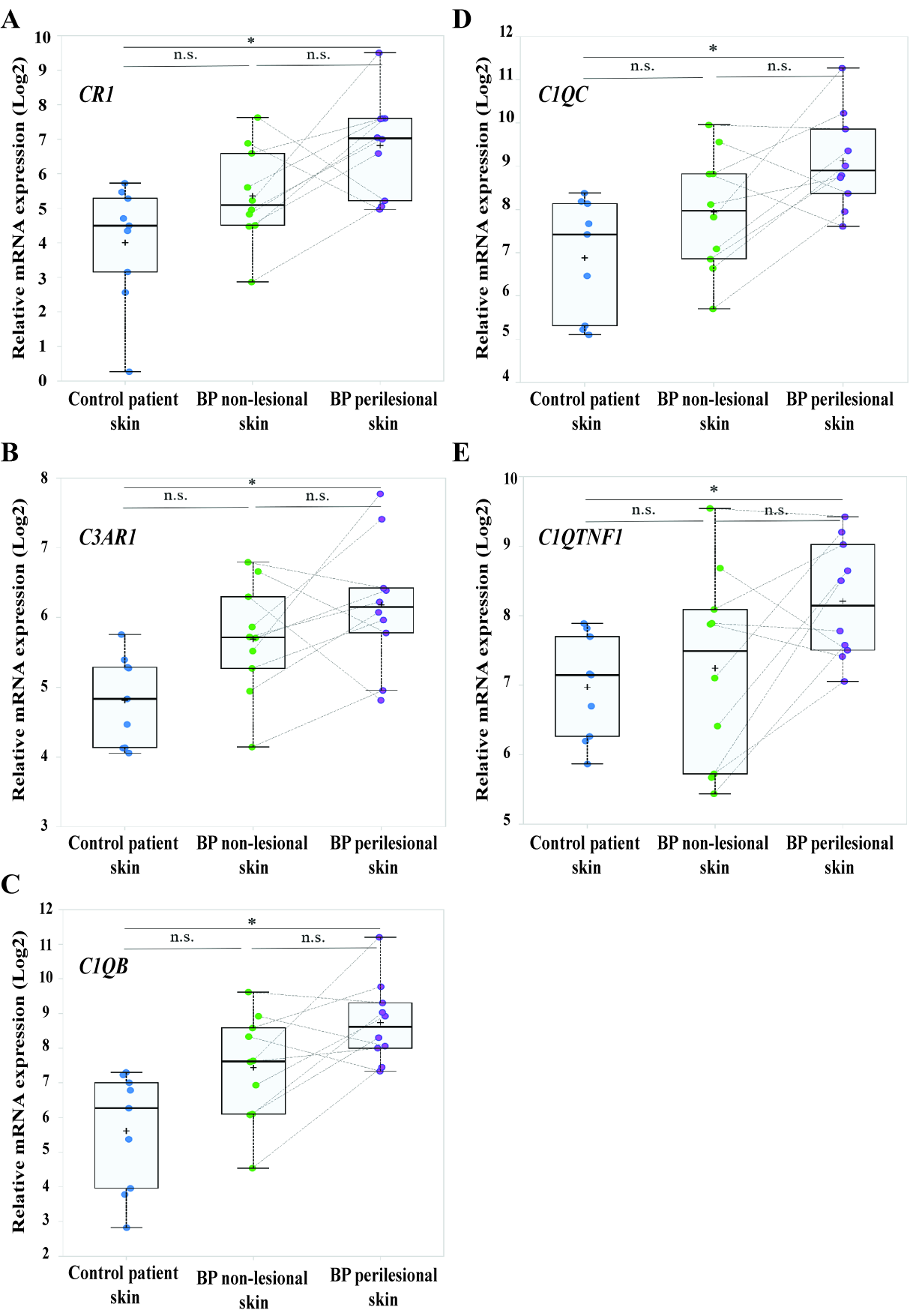

Supplement: Supplementary Figure 1 — mRNA levels of complement receptor and complement-associated genes are enhanced in early bullous pemphigoid (BP) skin lesions. (A-E) Dot-plot graphs depicting the relative RNA expression levels of differentially expressed genes, including CR1 (A), C3AR (B), C1QB (C), C1QC (D), and C1QTNF1 (E) between the three clinical groups: site-matched skin of age- and sex-matched controls (n=9), site-matched BP non-lesional skin (n=10), and BP perilesional skin (n=10). Plots were based on normalized and log2 transformed FPKM values and the identification of differentially expressed genes was conducted by DESeq2. FPKM, fragments per kilobase of transcript per million mapped reads; FDR, false discovery rate; *, FDR <0.05; n.s., not significant. [file Image_1.tif]

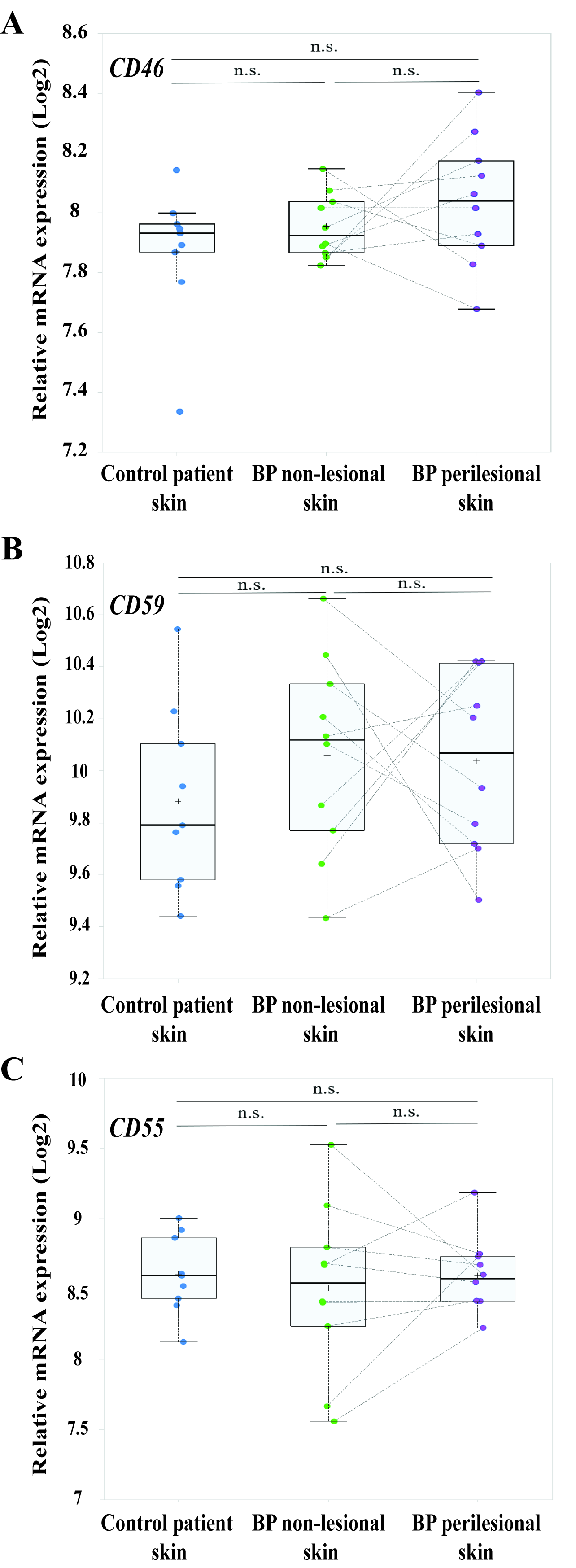

Supplement: Supplementary Figure 2 — mRNA levels of CD46, CD59, and CD55 are not significantly altered in early bullous pemphigoid (BP) skin lesions compared to both non-lesional BP skin and skin of controls. (A-C) Dot-plot graphs depicting the relative mRNA levels of complement regulatory genes, including CD46 (A), CD59 (B), and CD55 (C) between the three clinical groups: site-matched skin of age- and sex-matched controls (n=9), site-matched BP non-lesional skin (n=10), and BP perilesional skin (n=10). Plots were based on normalized and log2 transformed FPKM values and the identification of differentially expressed genes was conducted by DESeq2. FPKM, fragments per kilobase of transcript per million mapped reads; FDR, false discovery rate; *, FDR <0.05; n.s., not significant. [file Image_2.tif]

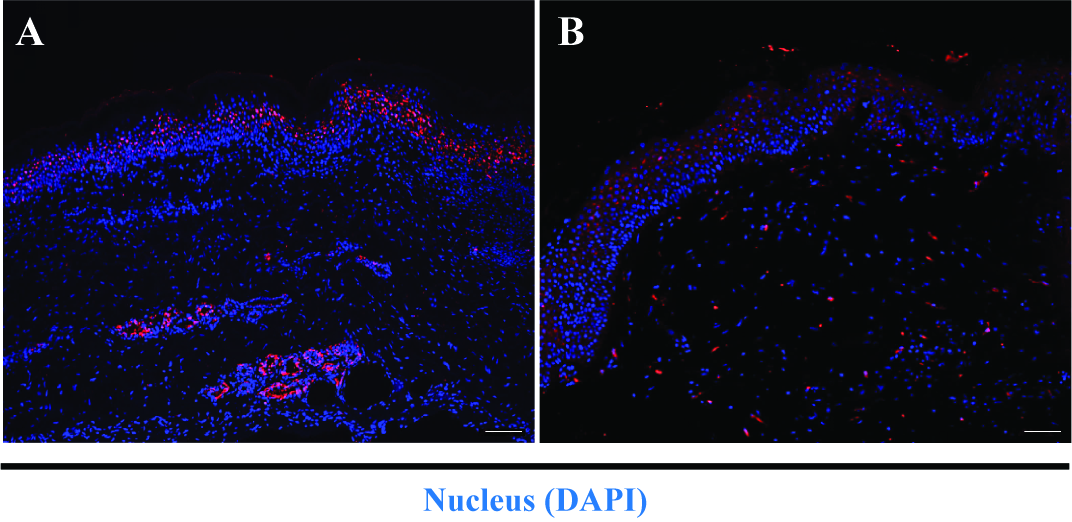

Supplement: Supplementary Figure 3 — Immunofluorescence staining showed expression of C5aR1 (A) and C5aR2 (B) on epithelial cells and vascular endothelial cells. DAPI staining of nuclei is shown in blue. Scale bars, 100 µm. [file Image_3.tif]

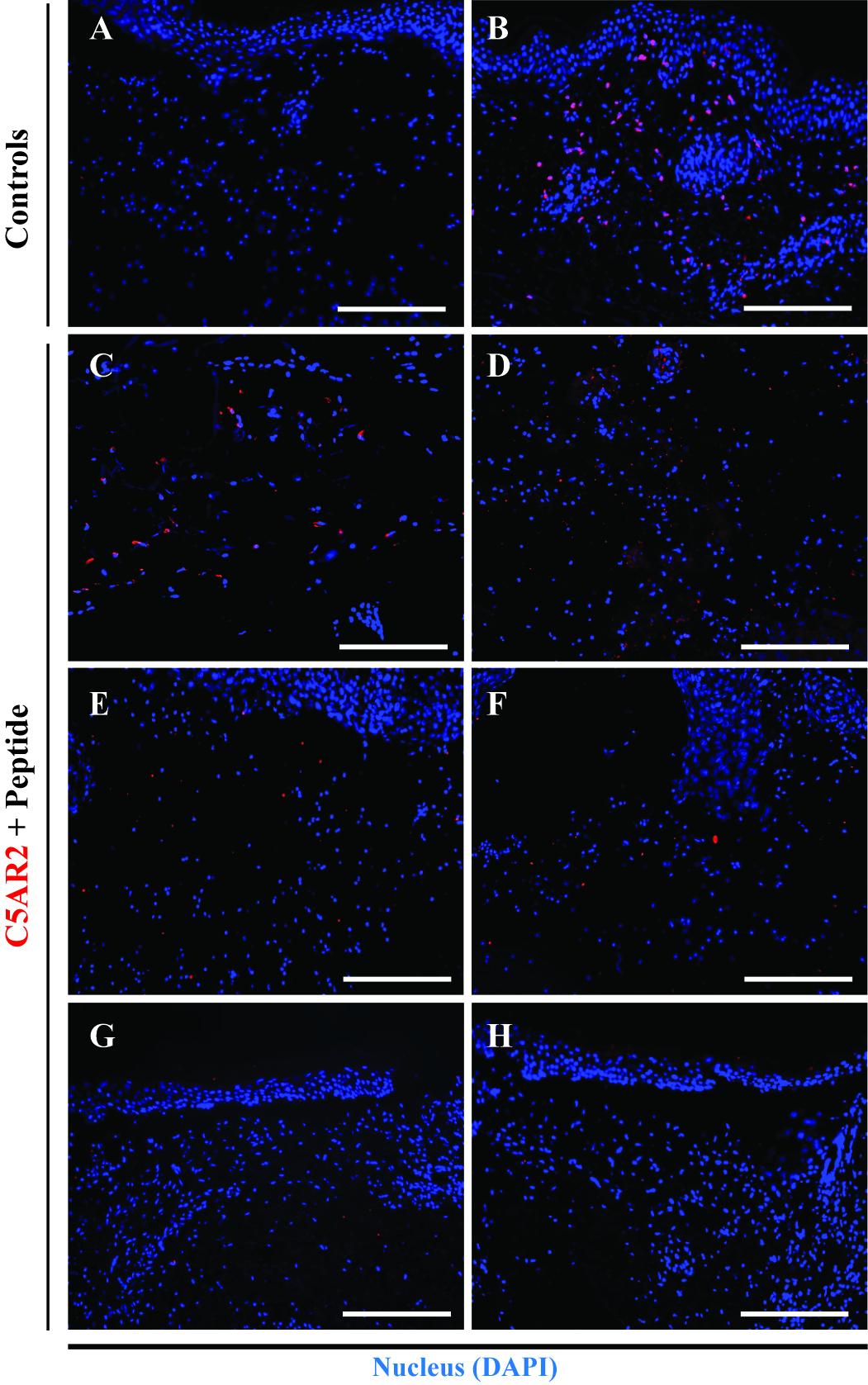

Supplement: Supplementary Figure 4 — The specificity of the anti-C5aR2 antibody was validated by coincubation with increasing amounts of a C5aR2 peptide. Sections of randomly selected BP skin were stained with isotype control antibody (A), anti-C5aR2 antibody alone (B), and anti-C5aR2 antibody plus increasing amounts of the C5aR2 peptide (RRLHQEHFPARLQCVVDYGGSSSTEN), i.e. 0.1 (C), 0.5 (D), 1 (E), 5 (F), 25 (G), and 50 µg (H), respectively. A dose-dependent inhibition of the IF staining was seen with the increasing C5aR2 peptide amounts indicating a high specificity of the anti-C5aR2 antibody. Incubation of the anti-C5aR2 antibody with 50 µg of an irrelevant control peptide did not alter the IF staining (data not shown). Data are representative of results obtained from three experiments. Scale bars, 100 µm. [file Image_4.tif]

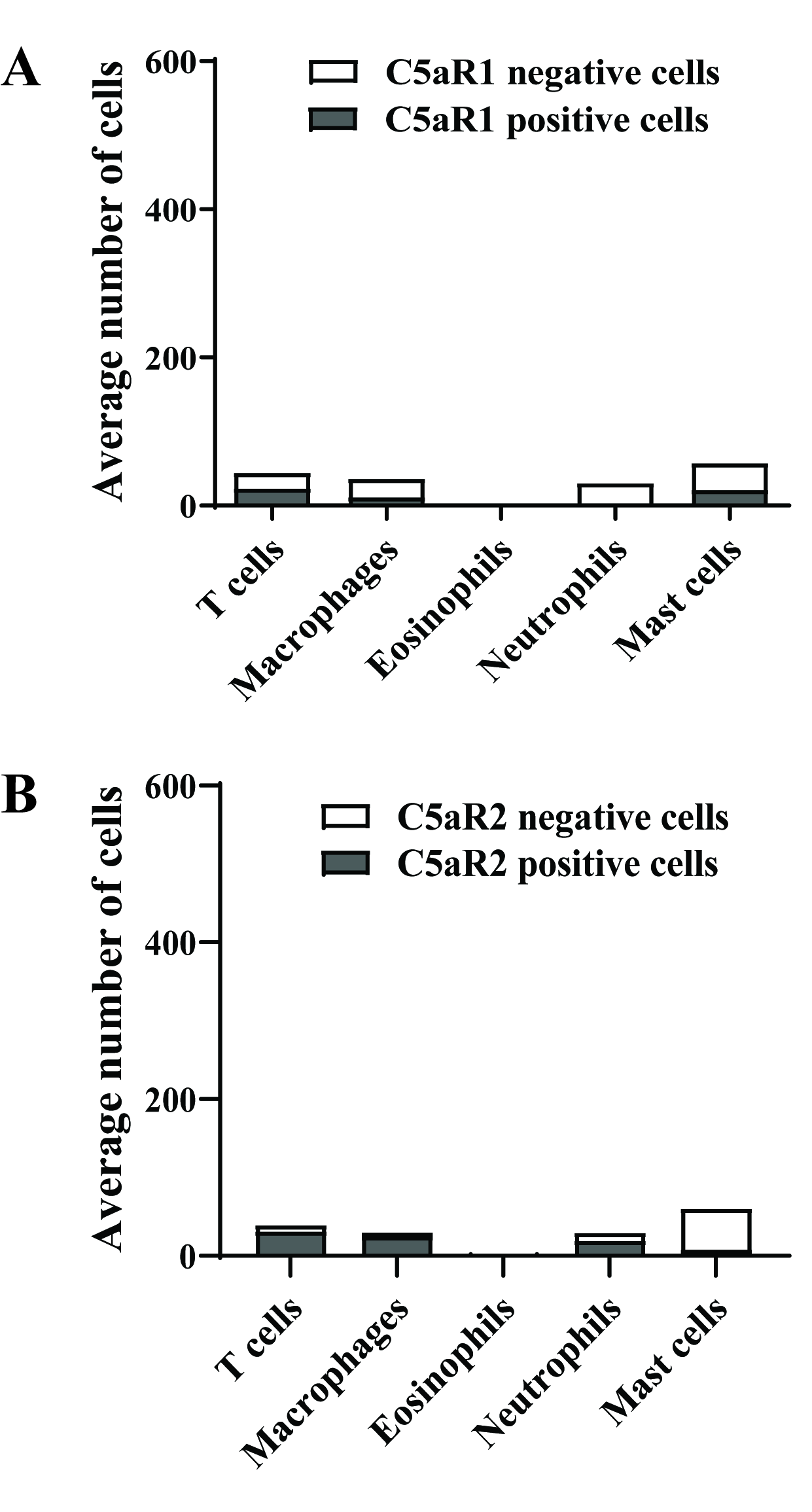

Supplement: Supplementary Figure 5 — In site-matched skin biopsies of age- and sex-matched control patients (n=4), only few inflammatory cells were detected with low expression of C5aR1 (A) and C5aR2 (B). [file Image_5.tif]

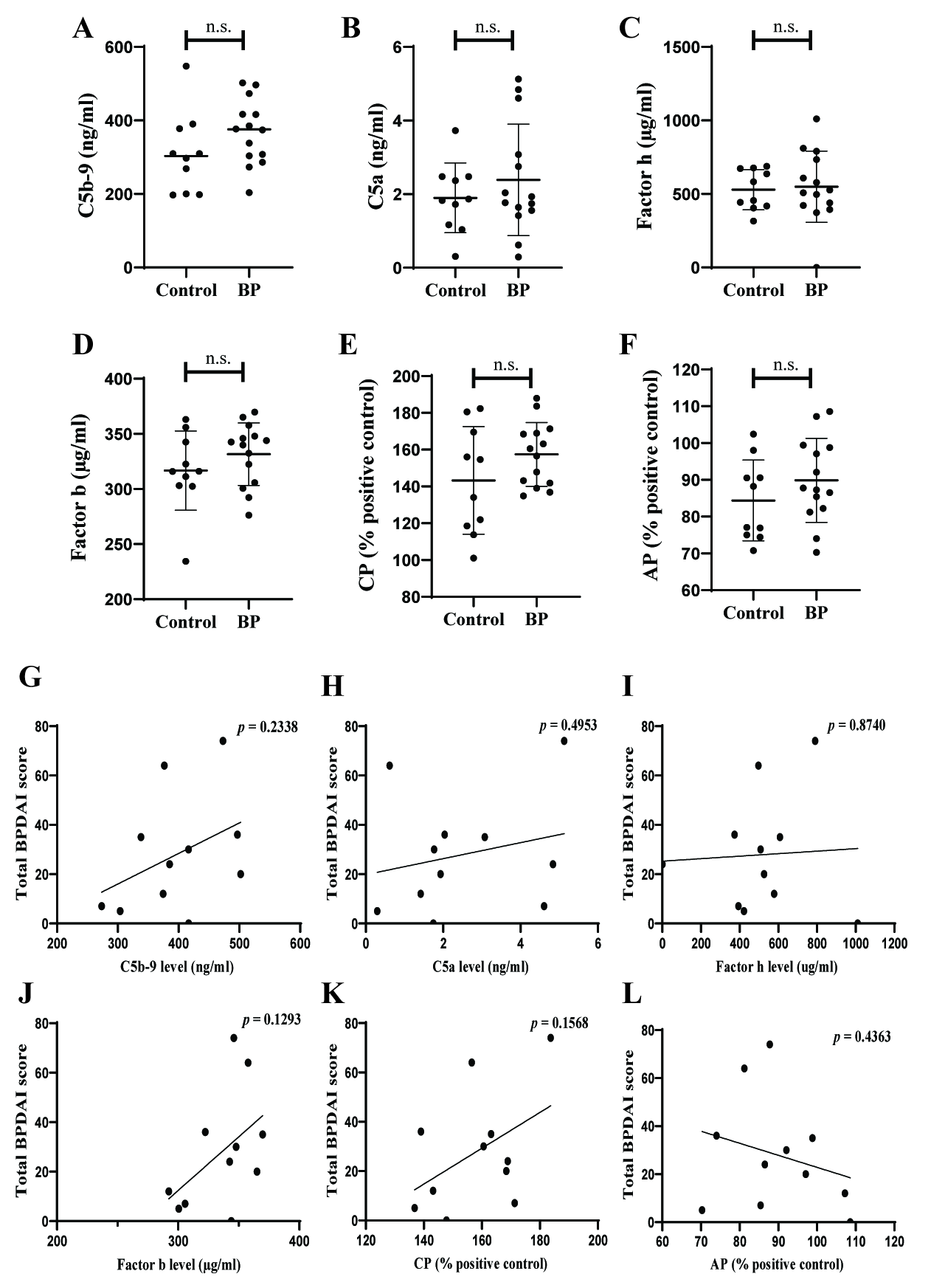

Supplement: Supplementary Figure 6 — Plasma levels of C5b-9, C5a, factor h, and factor b as well as serum activity of the classical (CP) and alternative complement (AP) pathways in patients with bullous pemphigoid (BP) and controls. (A-F) Plasma levels of C5b-9 (A), C5a (B), factor h (C), and factor b (D) as well as serum activity of the CP (E) and the AP (F) in BP patients (n=10) and age- and sex-matched controls (n=10) did not show significant differences. (G-L) Plasma levels of C5b-9 (G), C5a (H), factor h (I), and factor b (J) as well as serum activity of the CP (K) and the AP (L) in BP patients did not significantly correlate with diseases activity as measured by the bullous pemphigoid disease area index (BPDAI). Differences between groups were analyzed by unpaired two-tailed t-test. n.s., not significant. [file Image_6.tif]
